# Seed layer formation by deposition of micro-crystallites on a revolving substrate. Part II: application to some nonlinear effects

**DOI:** 10.1107/S205252062600764X

**Published:** 2026-09-01

**Authors:** Arthur Ballato, John Ballato

**Affiliations:** a Holcombe Department of Electrical and Computer Engineering Clemson University Clemson SC29634 USA; b Department of Materials Science and Engineering Clemson University Clemson SC29625 USA; University of Geneva, Switzerland

**Keywords:** deposition, sputtering, nonlinear properties, rotating substrate, crystallites, seed layer, texture, piezoelectricity, altermagnetism, cubic magneto-optic Kerr effect

## Abstract

The matrix method of averaging material coefficients of crystallites deposited at an angle to a revolving substrate, disclosed in the prequel [Ballato & Ballato (2024). *Acta Cryst.* B**80**, 760–765], is applied to tensors in their indicial form. The results are used to determine averaged nonlinear dielectric, piezoelectric, and elastic constants, as well as linear pyroelectric coefficients, for triclinic crystals; these are further specialized for Laue classes of higher symmetry.

## Abstract 2

A method of averaging the material coefficients of crystallites deposited at an angle onto a revolving substrate isapplied to effective nonlinear dielectric, piezoelectric and elastic constants, as well as linear pyroelectric coefficients, for triclinic crystals.

## 1. Introduction

In Part I (Ballato & Ballato, 2024 ▸), referred to subsequently as (I), a simple model was proposed for averaging material coefficients of crystallites deposited at an angle to a revolving substrate. It was implemented to determine the average linear dielectric permittivity, piezoelectric stress, and elastic stiffness matrices for all Laue classes, as functions of those of an unrotated single crystal. These particular coefficients were selected because they are employed in many electro-elastic applications (Weigel *et al.*, 2002 ▸), and serve as examples applied to tensorial rank 2, 3 and 4 effects (Bhagavantam & Suryanarayana, 1949 ▸; Smith, 1958 ▸; Huntington, 1958 ▸; Mason, 1966 ▸; Nye, 1985 ▸). Here the model is applied to some nonlinear versions of these quantities.

The phenomenology of recoverable electro–elastic interactions in materials in the continuum approximation is often expressed in the form of power series expansions of an energy function, yielding coefficients of various orders (Truesdell & Toupin, 1960 ▸). In many instances first-order estimations prove to be admirably sufficient for practical applications. The linear equations of piezo-elasticity are a notable example (Forsbergh, 1956 ▸; IEEE Standard on Piezoelectricity, 1987 ▸). Increasingly, however, departures from the linear regime have become important (Truesdell & Noll, 1965 ▸; Forsbergh, 1956 ▸; Gagnepain & Besson, 1975 ▸; Maugin, 1986 ▸; Hruška, 2014 ▸). In discussions of nonlinearities, however, one must distinguish the cases where cause–effect relations are strictly single valued and those that exhibit hysteresis, or history-dependent behavior (Guo *et al.*, 2025 ▸). This latter case is briefly mentioned in Section 5.5, where ferroelectric materials and linear pyro­electricity are discussed.

## 2. Rationale for this work

It has long been recognized that the thin film fabrication scenarios depicted in (I) give rise to strains at the heterostructure interfaces (Fujiwara *et al.*, 1986 ▸; Nishino, 1989 ▸; Jain *et al.*, 1997 ▸). Indeed, it is to be expected that any discontinuities in the elastic and electric fields at junctions might require consideration of nonlinearities. Such gradients arise, *e.g.* in superlattices (Wierzbicka *et al.*, 2025 ▸), in composites, where two or more sub-materials or phases are joined at the microscopic scale, but are macroscopically homogeneous (van Suchtelen, 1972 ▸; van den Boomgaard *et al.*, 1976*a* ▸; van den Boomgaard *et al.*, 1976*b* ▸), and in multiferroics, which are homogeneous on the microscopic scale (Newnham, 2005 ▸; Fiebig *et al.*, 2016 ▸).

Apart from configurations where macroscopic spatial discontinuities / gradients produce nonlinear effects, atomic-level anharmonicities associated with the Grüneisen equation of state (Grüneisen, 1912 ▸; Anderson, 2000 ▸) implicitly involve considerations of nonlinearity. Examples include thermal conductivity / heat transport (Casimir, 1938 ▸; Kittel, 1949 ▸; Iwanowski *et al.*, 2025 ▸), and optical nonlinearities (Shanshool *et al.*, 2026 ▸). Section 5 provides further particulars.

An additional application of nonlinearities is to computation of temperature coefficients of the elastic stiffnesses. There are two contributions to these anharmonicities: the first is the geometrical nonlinearity, comprised of products of linear thermoelastic coefficients and the linear elastic stiffnesses; this geometrical portion is closely related to Poisson’s ratio considerations (Ballato, 2010 ▸). The second contribution is the physical nonlinearity, comprised of products of linear thermo­elastic coefficients and nonlinear elastic stiffnesses (Lee *et al.*, 1975 ▸; Sorokin *et al.*, 1999 ▸; Sorokin & Telichko, 2011 ▸; Sorokin & Telichko, 2012 ▸). Temperature- and stress-insensitive crystal orientations for high-precision frequency control of oscillators have been realized by judicious compensation of elastic nonlinearities (Ballato, 1977 ▸).

## 3. Nonlinear terms identified

Many material tensors (of differing ranks) that characterize various physical interactions are enumerated, *e.g.* in Bhagavantam & Venkatarayudu (1962 ▸), Mason (1966 ▸), Nye (1985 ▸). The efficient matrix method used in (I) is limited to rank 4 and below (Auld, 1973 ▸), otherwise the full tensor expansion must be used (Bond, 1943 ▸). Three-dimensional tensors will in general have 3^rank^ components, but when indicial symmetries, required by a particular physical effect, are taken into account, the number of independent coefficients is reduced. The same tensor rank may as well correspond to a number of various effects, albeit with differing numbers of components. For example, the rank 4 tensor in Mindlin’s polarization gradient theory (Mindlin, 1968 ▸) relates six stress or strain variables with nine independent polarization gradients, so the 3^4^ = 81 rank 4 components are reduced in this instance to 54.

We adopt schematic versions of the series expansions of Gagnepain & Besson (1975 ▸) given on page 251 of that work, specifically their equations (20) and (21) to identify the terms to be evaluated. Similar expressions are considered by Lv *et al.* (2025 ▸). Terminology, symbols, and conventions are as given in (I) and by Ballato & Ballato (2023 ▸). For didactic purposes, tensor ranks are indicated by superscripts in parentheses.

Equation (20): 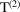 ∝ 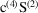 + 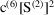 + 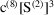 − 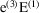 − 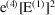 − 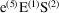 − 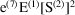 − 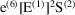 − 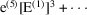.  The first and fourth terms were treated in (I).

Equation (21): 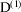 ∝ 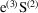 + 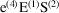 + 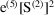 + 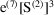 + 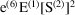 + 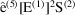 + 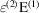 + 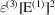 + 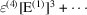. The first and seventh terms were treated in (I).

The lowest-order nonlinear portions of these equations are: T^(2)^ ∝ + c^(6)^[S^(2)^]^2^ − e^(4)^[E^(1)^]^2^ − e^(5)^E^(1)^S^(2)^ and D^(1)^ ∝ + e^(4)^E^(1)^S^(2)^ + e^(5)^[S^(2)^]^2^ + ɛ^(3)^[E^(1)^]^2^. The remaining terms represent higher-order (and usually insignificant) nonlinearities, and are disregarded for our purposes. We choose, as representative, the following nonlinearities:

Rank 3: dielectric permittivity tensor ɛ^(3)^ from D^(1)^ ∝ + ɛ^(3)^[E^(1)^]^2^

Rank 4: electrostriction tensor e^(4)^ from T^(2)^ ∝ − e^(4)^[E^(1)^]^2^ or D^(1)^ ∝ + e^(4)^E^(1)^S^(2)^

Rank 5: piezoelectric stress tensor e^(5)^ from T^(2)^ ∝ − e^(5)^E^(1)^S^(2)^ or D^(1)^ ∝ + e^(5)^[S^(2)^]^2^

Rank 6: elastic stiffness tensor c^(6)^ from T^(2)^ ∝ + c^(6)^[S^(2)^]^2^

In tensor notation these quantities appear, respectively, as: ɛ^(3)^ → ɛ_ijk_ → ɛ_iλ_; e^(4)^ → e_hijk_ → *e*_λμ_; e^(5)^ → e_kghij_ → e_kλμ_; and c^(6)^ → c_fghijk_ → c_λμν_. As usual, Roman indices range 1, 2, 3 and Greek (Voigt) indices range 1, 2, … 6.

The expansions given can also be expressed in the form of reciprocal relations. For example, the nonlinear elastic stiffnesses c^(6)^ have compliance counterparts s^(6)^ determined from the relation S_ijklmn_ = −S_ijpq_S_klrs_S_mnuv_C_pqrsuv_ (Ballato, 2001 ▸; Kube & Turner, 2016 ▸). Compliances are used to characterize the nonlinear behavior of thin rods and bars (Tiersten & Ballato, 1983 ▸).

## 4. The model

**Figure 1:**
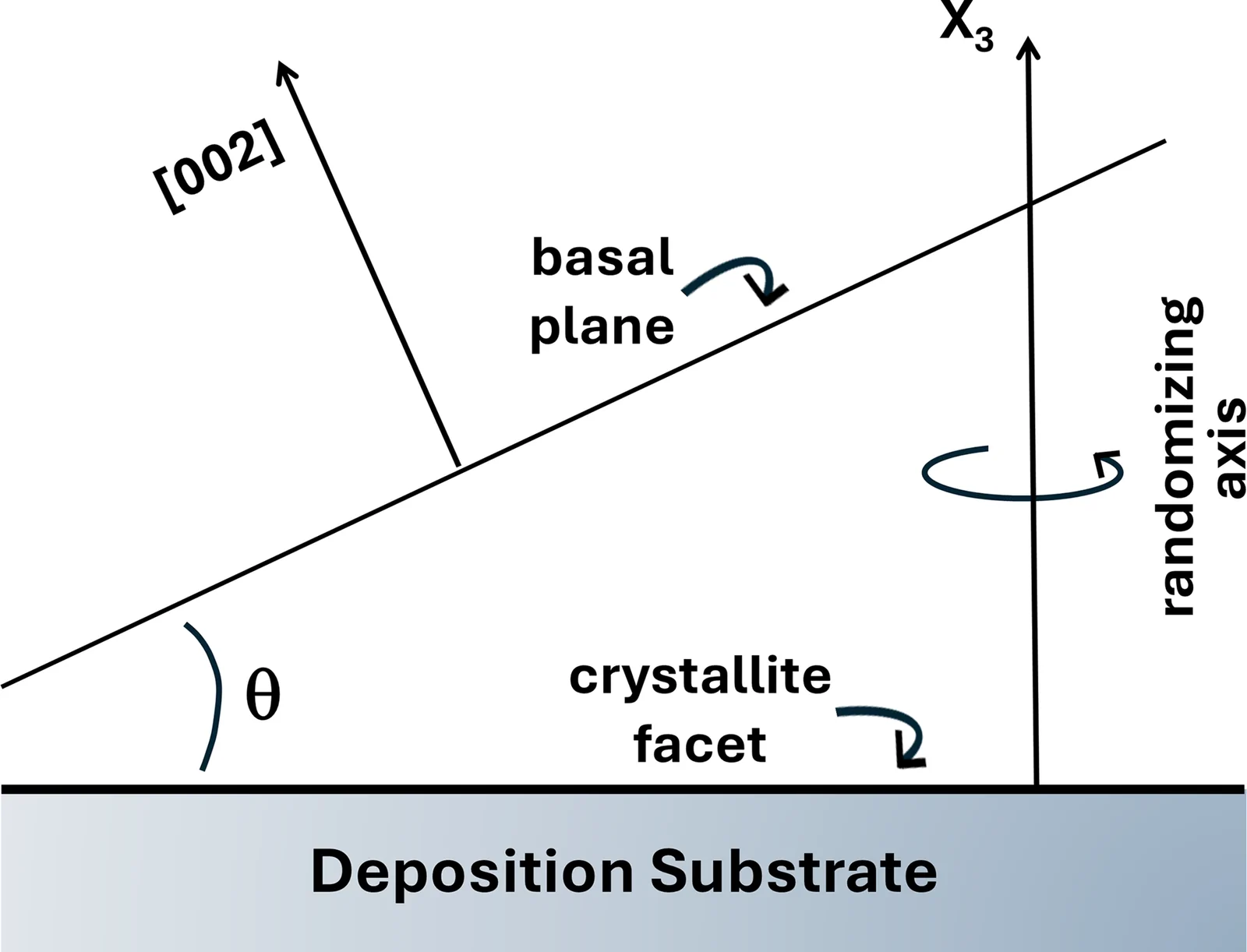
Schematic of the deposition geometry. The crystallite facet lies upon the substrate; its *x*_3_ axis, shown as [002], subtends cone angle θ with respect to the substrate randomizing axis *X*_3_.

Micro-crystallites are deposited on a revolving substrate. Prior to the deposition, in the reference state the crystallite *x*_3_ axis is taken to be normal to the substrate reference plane; a first rotation is then made about its *x*_1_ or *x*_2_ axis. Upon deposition, a specified facet is taken to lie on the substrate plane. The *x*_3_ axis of the crystallite is thus inclined with respect to the substrate normal (*X*_3_) by a facet angle θ; see Fig. 1 ▸.

As a result of the *x*_1_ or *x*_2_ 90° rotation, followed by the θ inclination, material coefficients (generically symbolized as *q*) that originally referred to crystallographic axes (IEEE Standard on Piezoelectricity, 1987 ▸) are transformed to new coefficients *q*′.

Subsequent rotation of the crystallite sitting on the substrate plane is described by a rotation, in angle φ, about the substrate *X*_3_ axis. This creates a distribution of crystallite normals ([002] in the diagram) that form a cone about the substrate normal. A suitable angular averaging then yields average values, applied to the individual tensor components, 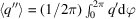, where *q*′ is the quantity to be averaged.

## 5. Model results ordered by rank and Laue group

In this section are given the averaged components, 〈′′〉, of the nonlinear coefficients selected above, ordered by rank and Laue group (Bechmann & Hearmon, 1969 ▸; Brendel, 1979 ▸). Ordering by rank avoids the confusion of terms such as first-order, third-order *etc*.

### 5.1. Rank 3, nonlinear dielectric permittivities, ɛ^(3)^

The nonlinear dielectric permittivity (ɛ_*ijk*_) is a tensor of rank 3, symmetric in the indices assigned to the two independent electric field directions. By converting these two indices into Voigt form, the 3^3^ = 27 components are reduced to 18 in the general Laue group I (triclinic) case. The outcome is isomorphic with the linear piezoelectric effect treated in (I). The averaging results may therefore be recovered from Section 3.2 of (I) for all Laue classes; in Table 5 thereof the coefficient of e_15_ should read C, not C^2^.

Under the conditions posited by Kleinman (1962*a* ▸, 1962*b* ▸) for optical second-harmonic generation (SHG), all three indices permute, and the 18 conditions are further reduced to 10, which we take as those with the Voigt indices: 11, 12, 13, 14, 15, 16, 22, 23, 24 and 33. The Kleinman indicial identities are: 21 = 16, 25 = 36 = 14, 26 = 12, 31 = 15, 32 = 24, 34 = 23 and 35 = 13. The hyperpolarizability tensor obeys the same relations; it is a molecular (microscopic) cause, whereas SHG is a collective (macroscopic) and observable effect arising therefrom.

Application of the averaging procedure, outlined in (I) to the SHG tensor yields the triclinic results given in Tables 1 ▸ and 2 ▸. The seven elements 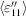, 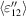, 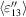, 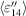, 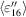, 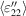 and 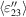 vanish identically for both *x*_1_ and *x*_2_ rotations, leaving only 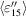, 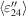 and 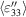, but 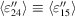, so there are but two SHG 〈ɛ′′〉 coefficients. Furthermore, the coefficients ɛ_11_, ɛ_12_, ɛ_14_ and ɛ_13_ do not appear in the expressions for 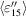 and 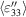.

Twelve point groups have finite SHG 〈*e*′′〉 values; these are given in Table 3 ▸. Classes 222, 422, 622, 42*m*, 32, 6*m*2, 23 and 43*m* are identically zero when 〈ɛ′′〉 averaging is applied, and the pairs (*m*, *mm*2), (4, 6), (3, 3*m*) and (4*mm*, 6*mm*) have the same form.

### 5.2. Rank 4, nonlinear piezoelectric stress (electrostriction) coefficients, *e*^(4)^

Electrostriction is a tensor of rank 4, relating mechanical deformations to imposition of an electric field. The effect is quadratic in the electric field; the e^(4)^ symbols appearing in the relations T^(2)^ ∝ −e^(4)^[E^(1)^]^2^ and D^(1)^ ∝ + e^(4)^E^(1)^S^(2)^ are identical (Hruška, 1965 ▸; Hruška, 2014 ▸).

Ferroelectric materials are briefly discussed in Section 5.5. A subset of these (relaxor ferroelectrics) (Cross, 1987 ▸) are a notable class that possess very high electrostriction and linear dielectric permittivities, with a variety of energy storage, harvesting and conversion applications.

Considering the relation T^(2)^ ∝ −e^(4)^[E^(1)^]^2^, the stress tensor is symmetric: T_jk_ = T_kj_, and the electric field products are indistinguishable: E_m_E_n_ = E_n_E_m_; however the stress and electric field indices cannot be reversed; λμ ≠ μλ. Therefore the 3^4^ = 81, rank 4, e^(4)^ components are reduced to 36 for triclinic symmetry, and not to 21 as in the elastic case when Voigt indices are used. When the averaging procedure is applied thereto, for either *x*_1_ or *x*_2_ rotation, the resulting triclinic 〈ɛ′′〉 contains 18 components, nine of which are independent; these are given in Table 4 ▸. It is similar in structure, but not identical, with the tetragonal electrostrictive matrix in Table 6 of Forsbergh (1956 ▸). Table 5 ▸, for the *x*_1_ rotation, expresses the 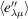 in terms of the 36 unrotated e^(4)^ coefficients. The corresponding results for the *x*_2_ rotation are given in Table 6 ▸.

When, in Table 5 ▸, it is further assumed that λμ = μλ, the coefficients 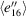 = 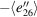 = 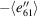 = 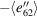 and 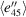 = 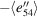 vanish. Additionally, 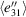 = 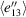. The resulting relations are those of the averaged linear elastic stiffnesses expressed in an alternative but equivalent form from those in Tables 7, 8, and 9 of (I).

### 5.3. Rank 5, nonlinear piezoelectric stress moduli *e*^(5)^

The rank 5 electroelastic effect, mentioned in Section 3 and discussed here, links the stress tensor with the product of electric field and strain tensors; reciprocally, it also links electric displacement with the product of the strain tensors. However, rank 5 phenomena encompass a wide variety of other physical effects including: stress dependence of the bulk photovoltaic effect (Nadupalli *et al.*, 2019 ▸), acoustical activity (Portigal & Burstein, 1968 ▸; Mindlin & Toupin, 1971 ▸; Frenzel *et al.*, 2019 ▸), cubic magneto-optic Kerr effect (Gaerner *et al.*, 2024 ▸) and altermagnetism (Krempaský *et al.*, 2024 ▸). Each of these disparate effects will require consideration of their individual point-group tensors; the commonality, for our purposes, is that they are all of rank 5.

Whereas the coefficient arrays for many material relationships depend solely on the Laue group (*e.g.* the linear and nonlinear elastic coefficients), the rank 5 arrays are different for each of the 21 permissible noncentrosymmetric (acentric) point groups, and all are listed in the admirable paper of Grimmer (2007 ▸). Halasyamani & Poeppelmeier (1998 ▸) provide a generous sampling of noncentrosymmetric (acentric) oxide representatives.

It is to be noted that IRE Standards on Piezoelectric Crystals (1949 ▸), Bechmann, (1953 ▸), Nelson & Hearmon (1979 ▸), Ballato & Ballato (2020 ▸) use *e*_*f*λ_ for the linear piezoelectric stress coefficient, and *d*_*f*λ_ for the linear piezoelectric strain coefficient; hence the corresponding nonlinear coefficients are denoted *e*_*f*λμ_ and *d*_*f*λμ_, respectively; Grimmer (2007 ▸) reverses these.

#### 5.3.1. Triclinic, Laue I, class 1

Whereas the number of rank 5 terms involves 3^5^ = 243 coefficients in general, elastic symmetries reduce these to 63 coefficients for Laue class I (three field directions times 21 elastic indices). When averaging is applied, only 25 survive; these are identical for both *x*_1_ and *x*_2_ rotations, and are: 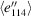, 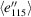, 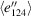, 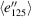, 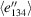, 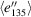, 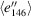, 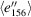, 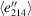, 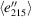, 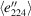, 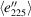, 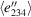, 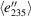, 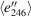, 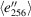, 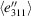, 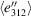, 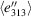, 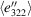, 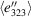, 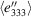, 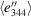, 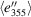 and 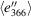. The number of these 25 surviving triclinic 〈*e*′′〉 coefficients will be further reduced depending on the additional symmetries pertaining to a particular effect.

The full expansion of each of the 25 surviving 〈*e*′′〉 coefficients will involve, in general, 63 terms for each rotation. As these are somewhat cumbersome, the full expansion for the triclinic case has been organized in the form of four tables for each rotation: for the *x*_1_ rotation, Tables 7 ▸, 8 ▸, 9 ▸ and 10 ▸, and for the *x*_2_ rotation, Tables 11 ▸, 12 ▸, 13 ▸ and 14 ▸. These are to be read in the generic form: 〈*e*′′〉 = *M*_1_*Z*_1_ + *M*_2_*Z*_2_ + *M*_3_*Z*_3_. The multipliers *M*_*k*_ are given in Tables 8 ▸ and 12 ▸; the quantities *Z*_*k*_ are found schematically in Tables 7 ▸ and 11 ▸, and written out in columns in Tables 9 ▸, 10 ▸, 13 ▸ and 14 ▸ in terms of the symbols *C* and *S*, which, in turn, stand for cos(θ) and sin(θ), where θ is the facet angle.

As an example, the expansion of 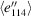 equals 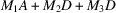, which in full reads:
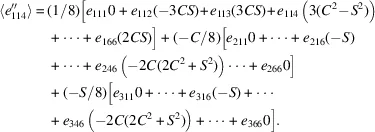
It will be seen that the majuscules (*x*_1_ rotation) and minuscules (*x*_2_ rotation) of the same alphabetic letter appearing in Tables 9 ▸, 10 ▸ and 13 ▸, 14 ▸ contain the same entries, albeit not in the same order, and with admixtures of sign reversals.

The 25 surviving triclinic 〈*e*′′〉 coefficients are further reduced in number when particularized for classes of higher symmetry. Classes 1[63], 4[15], 4[14], 3[21], 6[11], and 6[10] retain all 25 〈*e*′′〉 coefficients. Classes *m*[34], *mm*2[17], 4*mm*[10], 3*m*[13], 6*mm*[8], and 6*m*2[5] are diminished to 17 〈*e*′′〉 survivors: 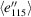, 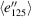, 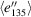, 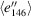, 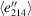, 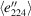, 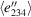, 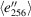, 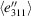, 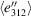, 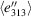, 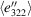, 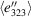, 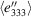, 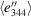, 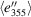 and 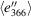. Classes 2[29], 222[12], 422[5], 42*m*[7], 32[8], 622[3], 23[4] and 432[1] are further diminished to eight survivors: 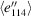, 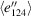, 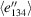, 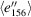, 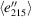, 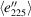, 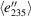 and 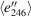. Class 43*m*[3] has no surviving coefficients. In square brackets above are the numbers of independent rank 5 coefficients for each point group (Grimmer, 2007 ▸). Given below are results for four classes of importance for micro- and nano-electronics.

#### 5.3.2. Hexagonal, Laue VIb, class 6*mm*

Class 6*mm* possesses eight independent *e*_fλμ_: *e*_115_ = *e*_224_, *e*_125_ = *e*_214_, *e*_135_ = *e*_234_, *e*_311_ = *e*_322_, *e*_312_, *e*_313_ = *e*_323_, *e*_333_ and *e*_344_ = *e*_355_, with auxiliary relations *e*_146_ = *e*_256_ = (1/2)(*e*_115_ − *e*_125_), and *e*_366_ = *e*_256_ = (1/2)(*e*_311_ − *e*_312_). Each of the 17 〈*e*′′〉 results are the same for both *x*_1_ and *x*_2_ rotations and are given in Table 15 ▸.

#### 5.3.3. Cubic, Laue VIIa, class 23

The four independent *e*_fλμ_ of class 23 are: *e*_114_ = *e*_225_ = *e*_336_, *e*_124_ = *e*_235_ = *e*_316_, *e*_134_ = *e*_215_ = *e*_326_, and *e*_156_ = *e*_246_ = *e*_345_. The eight 〈*e*′′〉 survivors of class 23 are identical with the vanishing members of class 6*mm*, but with the further relations: 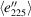 = 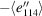, 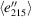 = 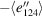, 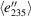 = 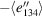, and 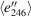 = 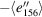, for both rotations. The final results are: 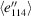 = 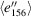 = 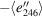 = 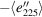 = 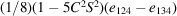, 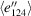 = 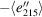 = 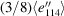 and 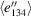 = 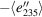 =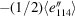. The *x*_1_ relations are converted to those for *x*_2_, by the substitution: *e*_124_ → *e*_134_.

#### 5.3.4. Cubic, Laue VIIb, class 432

This class is included here because its linear piezoelectric effect is identically zero; the nonlinear *e*_fλμ_ provide a driving mechanism for future applications of materials in this class. This class has but one independent *e*_fλμ_: *e*_124_ = −*e*_134_ = −*e*_215_ = *e*_235_ = *e*_316_ = −*e*_326_. After averaging, both *x*_1_ and *x*_2_ rotations give identical results for the eight remaining 〈*e*′′〉. Furthermore, these are multiples of 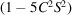 as follows: 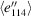 = 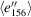 = 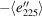 = 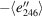 = 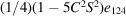; and 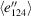 = 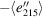 = 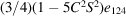; and 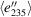 = 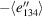 = 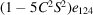. These results follow from those of class 23 by setting *e*_124_ = −*e*_134_ therein.

#### 5.3.5. Cubic, Laue VIIb, class 43*m*

This class possesses three independent *e*_fλμ_: *e*_114_ = *e*_225_ = *e*_336_; *e*_124_ = *e*_134_ = *e*_215_ = *e*_235_ = *e*_316_ = *e*_326_; *e*_156_ = *e*_246_ = *e*_345_. After averaging all 〈*e*′′〉 are zero; the rank 5 nonlinear piezo effect vanishes in class 43*m*.

### 5.4. Rank 6, nonlinear elastic stiffnesses, c^(6)^

A lucid paper by Murnaghan (1937 ▸) began the study of nonlinear elasticity. He treated isotropic solids, for which, to lowest order, three nonlinear coefficients suffice. This was extended to cubic solids by Birch (1947 ▸). Subsequently, many other treatments have followed, *e.g.* Thurston & Brugger (1964 ▸) and Brugger (1964 ▸); see also the references in Truesdell & Toupin (1960 ▸), Truesdell & Noll (1965 ▸) and Norris (2024 ▸). Among the contributors to this field should be noted those of Mindlin (Herrmann, 1974 ▸; Mindlin, 1989 ▸) and those of what might be called the ‘school of Mindlin’, *e.g.* Tiersten (1969 ▸), Baumhauer & Tiersten (1973 ▸), Lee *et al.* (1975 ▸), di Lorenzi & Tiersten (1975 ▸), Ancona & Tiersten (1980 ▸), Sinha (2001 ▸), Patel & Sinha (2015 ▸), Sinha & Wendt (2015 ▸). The study of nonlinear elasticity might have begun primarily as an academic exercise, but the results have now become of importance in a variety of disparate fields, such as determinations of thermal expansion (Sheard, 1958 ▸), light diffraction by sound waves (Melngailis *et al.*, 1963 ▸), lattice theories of crystals (Srinivasan, 1966 ▸), quartz resonator thermal and stress sensitivity compensation (Ballato, 1977 ▸), acoustoelastic characterization of materials (non-destructive evaluation) (Cantrell & Salama, 1991 ▸; Cantrell, 1994 ▸), Poisson’s ratio considerations (Ballato, 2010 ▸), calculations of textured polycrystals (Kube & Turner, 2016 ▸), and seismology (Stern *et al.*, 2017 ▸).

Rank 6 tensors have 3^6^ = 729 coefficients in general, but the third-order nonlinear elastic coefficients c^(6)^ for a triclinic crystal are reduced to 56 because the coefficients are symmetric in all three Voigt indices; the number of multiplicities of each coefficient are listed in Table 5, column 1 of Hearmon (1953 ▸). Whereas each of the 21 non-centrosymmetric point groups has a unique rank 5 piezoelectric matrix, the nonlinear elastic matrices are determined solely by the Laue group. In this sense, treating the rank 6 nonlinear elastic situation is somewhat simpler than that of rank 5 effects.

Fumi (1951 ▸, 1952*a* ▸, 1952*b* ▸) was the first to enumerate, accurately, these independent elastic coefficients for triclinic crystals; we hereby address his lament (Fumi, 1987 ▸). Brugger’s much more cited and comprehensive paper (Brugger, 1965) ▸ on the subject contains, in Table 3, the inter-relationships between certain of the *c*_λμν_, albeit in a footnote with a number of entries that are typographically ambiguous. Some of these may be recovered from Sirotin & Shaskolskaya (1982 ▸). All are listed in Table 16 ▸. Table 17 ▸ provides the number of independent elastic coefficients, of various orders, as function of the Laue classes. When the 56 independent rank 6 elastic coefficients are subjected to the averaging procedure, the result is a reduction to 24 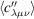 coefficients. The indices of these are given in Table 18 ▸. Table 19 ▸ provides the number of rank 6 elastic coefficients for all Laue groups, as well as those that are independent, and those that survive the averaging procedure.

Tables 20 ▸ ▸ ▸ ▸ ▸ ▸ ▸ ▸ ▸ ▸ ▸ ▸ ▸ ▸ ▸ ▸ ▸ ▸ ▸ ▸ ▸ ▸ ▸ ▸–43 ▸ provide the *x*_1_ rotation results for these 〈*c*′′〉, factored as powers of *C*^*n*^. In the even-order ranks, terms such as (*C*^2*n*^ + *S*^2*n*^) occur. These may always be reduced to a finite polynomial in (*C*^2^*S*^2^). The polynomial appears in the form 1 − *n*(*C*^2^*S*^2^) + *R*(*C*^2^S^2^), where *R*(*C*^2^*S*^2^) is a finite polynomial with all positive coefficients, and of order no greater than *n*/2. For example, because (*C*^2^ + *S*^2^) = 1, it follows that (*C*^2^ + *S*^2^)^2^ = 1 − 2(*C*^2^*S*^2^); (*C*^2^ + *S*^2^)^3^ = 1 − 3(*C*^2^*S*^2^), *etc*. However, with the exception of the results for cubic class 43*m* given subsequently, it appears simpler to display the results factored as powers of *C*^*n*^. Note that the table entries are to be divided by 16; that is, the expressions are to be read as 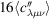 = [ ]*C*^6^ + [ ]*C*^5^ + ⋯ + [ ]*C*^0^. For nano- and micro-electronic application, the most useful results are those in hexagonal class 6*mm* (Laue VIb), and cubic class 43*m* (Laue VIIb).

Hexagonal, Laue VIb, class 6*mm* has ten independent coefficients: *c*_111_, *c*_112_, *c*_113_, *c*_123_, *c*_133_, *c*_144_, *c*_155_, *c*_222_, *c*_333_ and *c*_344_. Table 44 ▸ contains the 20 extant 6*mm*, *x*_1_ rotation, 〈*c*′′〉 coefficients in terms of these. The table is split into two parts for reasons of formatting, and is to be read as 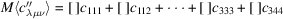. In this expression the multiplier *M* = 16, except it is 32 for 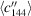, 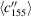 = 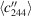, 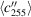 and 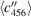. For each *c*_λμν_, the table entries, given schematically as square brackets above, contain four numbers, (*a*_*n*_), in the expansion [*a*_*n*_cos^2*n*^(θ)], where *n* = 3, 2, 1, 0. Taking 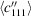 = 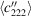 as an example, the expansion reads: 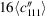 = 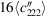 = 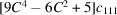 + 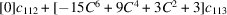 + 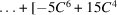 − 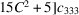 + 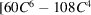 + 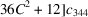, where *C* = cos(θ), and θ is the facet angle.

Cubic (Laue VIIb) class 43*m* has six independent coefficients: *c*_111_, *c*_112_, *c*_123_, *c*_144_, *c*_155_ and *c*_456_. In Table 45 ▸ are given the 20 surviving 43*m*, *x*_1_ rotation, 〈*c*′′〉 coefficients, formatted with two values for each *c*_λμν_, an integer plus the coefficient of (*CS*)^2^.

All the rank 6, *x*_2_ rotation formulas may be obtained from those for *x*_1_ rotation simply by making the following substitutions of the *c*_λμν_ in Tables 20 ▸–45 ▸: 1 → 2, 2 → 1, 3 → 3, 4 → −5, 5 → 4 and 6 → −6.

### 5.5. Ferroelectricity: a different type of nonlinearity

Of the 32 crystallographic point groups, 21 are noncentrosymmetric (acentric). Fig. 1 ▸ in Halasyamani & Poeppelmeier (1998 ▸) provides an insightful Venn diagram showing various property interrelationships between these noncentrosymmetric (acentric) point groups. With the exception of class 432, all noncentrosymmetric (acentric) groups allow linear piezoelectricity. The 20 piezoelectric classes are further divided according to whether they do, or do not, possess a spontaneous electric dipole moment, *i.e.* are polar or non-polar. Materials in the ten polar classes exhibit pyroelectricity, a rank 1 (vectorial) effect relating charge production and temperature change (Chynoweth, 1956 ▸; Mason, 1971 ▸; Lang, 2005 ▸). The pyroelectrics are further subdivided into those whose electric polarization is, or is not, reversible upon imposition of a sufficiently strong electric field. Those allowing reversible polarization are called ferroelectrics. The relationship between polarization and the applied electric field describes a hysteresis loop, so the polarization depends on the material’s previous history, and thus this nonlinearity is not readily amenable to a power series formulation like the other recoverable effects treated herein. Another example of nonlinear hysteretic behavior occurs with coupled elastic modes in finite piezoelectric resonators, where so-called ‘activity dips’ occur (Li *et al.*, 2026 ▸).

Discovery of the ferroelectric hysteretic effect is attributed to Valasek (1920 ▸, 1921 ▸, 1922 ▸, 1971 ▸) by Jaffe (1964 ▸, 1975 ▸). Jaffe also discusses unrecovered, classified reports, dated 1918, to the National Research Council by J. A. Anderson (Stanford University) and W. G. Cady (Wesleyan University) that he had seen; the contents of these reports, if subsequently recovered, would furnish material for a lively debate regarding priority. From his personal interactions with Jaffe, one author (AB), has no doubt as to the existence and contents of the reports.

### 5.6. Linear pyroelectric coefficients

Soon after the discovery of this effect, it was found experimentally that those few ferroelectric materials then known had much larger linear pyroelectric coefficients than ordinary polar crystals. This spurred a search for additional ferroelectrics, and determinations of their phenomenological elastic, piezoelectric, dielectric, and pyroelectric material coefficients (Shirane *et al.*, 1955 ▸; Pepinsky *et al.*, 1956 ▸; Pepinsky & Jona, 1957 ▸). The suitability of materials such as triglycine sulfate (TGS, monoclinic space group *P*2_1_) for thermal devices utilizing the pyroelectric effect was recognized in the early 1960s (Hoshino *et al.*, 1957 ▸; Konstantinova *et al.*, 1960 ▸; Ballato, 1961 ▸). Since then the gamut of useful pyroelectric materials has dramatically increased (Srinivasan, 1984 ▸; Lang, 2005 ▸), although TGS still remains the material of choice for many thermal applications such as in sensors / detectors (temperature measurement, UV/IR detection, motion sensing, spectrometry), energy harvesters, and particularly in thermal imagers (medical diagnostics, building inspection, surveillance) (Velarde *et al.*, 2021 ▸).

The pyroelectric vector arrays are given in Table 46 ▸ for the ten crystal classes in which this effect can exist. Table 47 ▸ displays the results resulting from the averaging procedure, for both *x*_1_ and *x*_2_ rotations. It is worth noting that these results are identical for the electrocaloric effect (Forsbergh, 1956 ▸; Newnham, 2005 ▸) and for the pyromagnetic effect (Chynoweth, 1958 ▸).

## 6. Conclusions

The rotating substrate method of crystallite deposition applied in the prequel to linear material coefficients of ranks 2, 3 and 4, has been extended to certain nonlinear effects characterized by coefficients of ranks 3, 4, 5 and 6. These include general triclinic versions of dielectric, piezoelectric, and elastic effects, as well as linear, rank 1, pyroelectricity.

## Figures and Tables

**Table 1:** Rank 3 averaged nonlinear dielectric permittivities for triclinic Laue group I, with first rotation *x*_1_ Optical second-harmonic generation (SHG) conditions of Kleinman (1962*a* ▸, 1962*b* ▸) apply. *C* and *S* are cos(θ) and sin(θ), respectively, where θ is the facet angle.

ɛ_*i*λ_ →	ɛ_16_	ɛ_15_	ɛ_22_	ɛ_24_	ɛ_23_	ɛ_33_
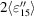	−*S*	*C*	−*C*^2^*S*	*C*(*C*^2^ − 2*S*^2^)	−*S*(*S*^2^ − 2*C*^2^)	*CS* ^2^
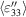			−*S*^3^	3*CS*^2^	−3*C*^2^*S*	*C* ^3^

**Table 2:** Rank 3 averaged nonlinear dielectric permittivities for triclinic Laue group I, with first rotation *x*_2_ Optical SHG conditions of Kleinman (1962*a* ▸, 1962*b* ▸) apply. *C* and *S* are cos(θ) and sin(θ), respectively, where θ is the facet angle.

ɛ_*i*λ_ →	ɛ_16_	ɛ_15_	ɛ_22_	ɛ_24_	ɛ_23_	ɛ_33_
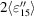	*S*	*C*	*C* ^2^ *S*	*C*(*C*^2^ − 2*S*^2^)	2*S*(*S*^2^ − 2*C*^2^)	*C* *S* ^2^
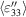			*S* ^3^	3*CS*^2^	−3*C*^2^*S*	*C* ^3^

**Table 3:** Specialization of averaged SHG nonlinear dielectric permittivities 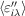 for all Laue groups having finite entries To be used in conjunction with Table 1 ▸ (*x*_1_ rotation) and Table 2 ▸ (*x*_2_ rotation); only *i*λ indices are tabulated.

Laue group	Point group	*i*λ indices					
I	1	15	16	22	23	24	33
II	2	0	16	22	23	0	0
II, III	*m*, *mm*2	15	0	0	0	24	33
IVa, VIa	4, 6	15	0	0	0	15	33
IVa	4	15	0	0	0	−15	0
Va, Vb	3, 3*m*	15	−22	22	0	15	33
VIa	6	0	−22	22	0	0	0
IVb, VIb	4*mm*, 6*mm*	15	0	0	0	15	33

**Table 4:** Rank 4, averaged, nonlinear piezoelectric stress (electrostriction) coefficient matrix for Laue group I The form of this matrix is the same for both *x*_1_ and *x*_2_ rotations, while the differences in the individual elements are given in Tables 5 ▸ and 6 ▸.

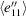	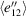	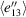	0	0	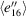
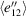	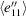	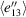	0	0	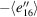
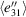	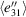	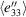	0	0	0
0	0	0	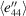	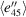	0
0	0	0	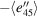	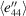	0
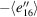	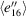	0	0	0	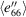

**Table 5:** The nine independent, averaged, rank 4 electrostriction coefficients 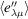 for Laue group I In terms of the 36 unrotated *e*_λμ_ values; *x*_1_ rotation; see Table 4 ▸. Entries are to be read as: 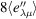 = *C*^4^[ ] + *C*^3^[ ] + *C*^2^[ ] + *C*[ ] + [ ]. *C* and *S* are cos(θ) and sin(θ), respectively, where θ is the facet angle.

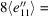	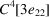	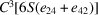	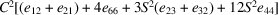
		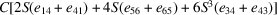	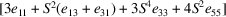

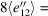	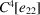	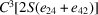	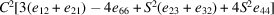
		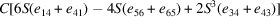	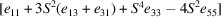

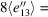	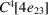	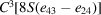	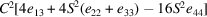
		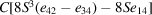	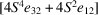

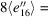	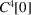	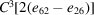	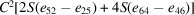
		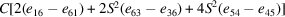	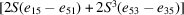

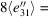	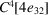	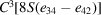	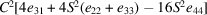
		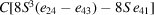	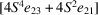

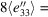	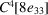	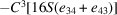	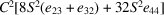
		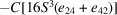	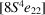

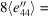	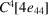	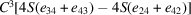	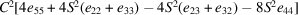
		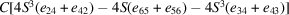	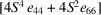

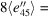	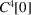	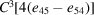	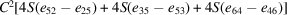
		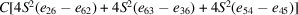	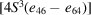

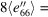	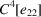	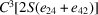	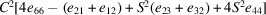
		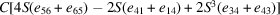	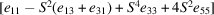

**Table 6:** The nine independent, averaged, rank 4 electrostriction coefficients 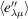 for Laue group I In terms of the 36 unrotated *e*_λμ_ values; *x*_2_ rotation; see Table 4 ▸. Entries are to be read as: 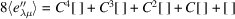. *C* and *S* are cos(θ) and sin(θ), respectively, where θ is the facet angle.

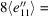	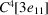	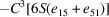	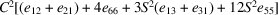
		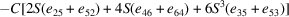	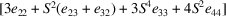

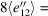	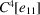	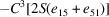	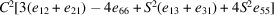
		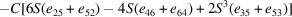	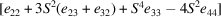

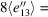	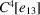	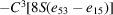	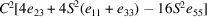
		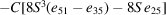	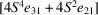

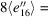	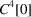	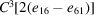	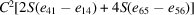
		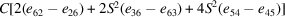	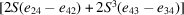

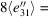	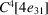	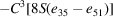	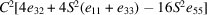
		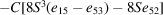	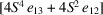

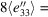	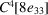	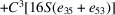	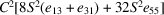
		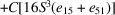	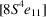

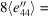	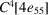	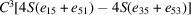	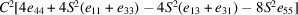
		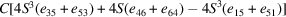	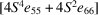

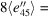	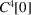	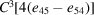	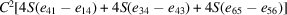
		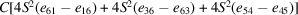	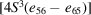

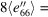	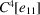	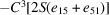	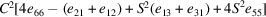
		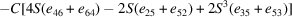	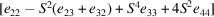

**Table:** This table is used in conjunction with Tables 8 ▸, 9 ▸ and 10 ▸; see text for details. For each of the nine 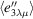, all *e*_1λμ_ components vanish for the *x*_1_ rotation. Apart from the multiplier, the *e*_2λμ_ and *e*_3λμ_ entries, for the *x*_1_ rotation, are identical for all 25 of the 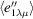, 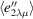 and 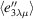.

*x*_1_ rotation	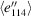	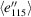	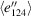	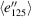	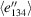	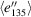	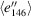	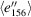
*e*_1λμ_	A	B	C	D	E	F	G	H
*e*_2λμ_ and *e*_3λμ_	D	C	B	A	F	E	H	G

**Table:** 

	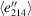	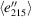	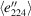	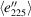	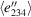	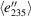	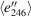	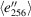
*e*_1λμ_	D	C	B	A	F	E	H	G
*e*_2λμ_ and *e*_3λμ_	A	B	C	D	E	F	G	H

**Table:** 

	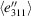	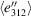	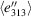	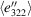	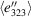	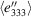	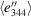	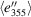	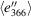
*e*_1λμ_	Zero								
*e*_2λμ_ and *e*_3λμ_	I	J	K	I	K	N	P	P	Q

**Table:** See text for details. Subscript f is the field direction.

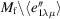	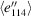	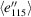	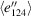	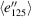	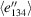	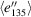	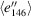	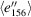
*M*_1_	1/8	1/8	1/8	1/8	1/2	1/2	1/8	1/8
*M*_2_	−*C*/8	*C*/8	−*C*/8	*C*/8	−*C*/2	*C*/2	*C*/8	−*C*/8
*M*_3_	−*S*/8	*S*/8	−*S*/8	*S*/8	−*S*/2	*S*/2	*S*/8	−*S*/8

**Table:** 

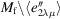	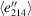	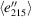	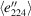	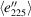	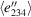	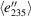	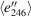	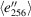
*M*_1_	1/8	−1/8	1/8	−1/8	1/2	−1/2	−1/8	1/8
*M*_2_	*C*/8	*C*/8	*C*/8	*C*/8	*C*/2	*C*/2	*C*/8	*C*/8
*M*_3_	*S*/8	*S*/8	*S*/8	*S*/8	*S*/2	*S*/2	*S*/8	*S*/8

**Table:** 

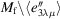	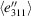	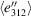	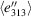	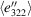	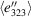	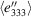	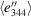	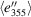	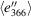
*M*_1_	Zero								
*M*_2_	−*S*/8	−*S*/8	−*S*/2	−*S*/8	−*S*/2	−*S*	−*S*/2	−*S*/2	−*S*/8
*M*_3_	*C*/8	*C*/8	*C*/2	*C*/8	*C*/2	*C*	*C*/2	*C*/2	*C*/8

**Table 9:** Expansions of the matrices A to H in terms of the unrotated *e*_*f*λμ_ Subscript f is the field direction. *C* and *S* stand for 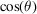 and 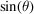, respectively, where θ is the facet angle.

	A	B	C	D	E	F	G	H
*e*_f11_	0	0	0	0	0	0	0	0
*e*_f12_	−3*CS*	0	−*CS*	0	0	0	0	*CS*
*e*_f13_	3*CS*	0	*CS*	0	0	0	0	−*CS*
*e*_f14_	3(*C*^2^ − *S*^2^)	0	(*C*^2^ − *S*^2^)	0	0	0	0	−(*C*^2^ − *S*^2^)
*e*_f15_	0	3*C*	0	*C*	0	0	*C*	0
*e*_f16_	0	−3*S*	0	−*S*	0	0	−*S*	0
*e*_f22_	−(*C*^3^*S*)	0	−(3*C*^3^*S*)	0	−*CS*^3^	0	0	−*C*^3^*S*
*e*_f23_	*CS*(*C*^2^ − *S*^2^)	0	3*CS*(*C*^2^ − *S*^2^)	0	−*CS*(*C*^2^ − *S*^2^)	0	0	*CS*(*C*^2^ − *S*^2^)
*e*_f24_	*C*^2^(*C*^2^ − 3*S*^2^)	0	3*C*^2^(*C*^2^ − 3*S*^2^)	0	*S*^2^(3*C*^2^ − *S*^2^)	0	0	*C*^2^(*C*^2^ − 3*S*^2^)
*e*_f25_	0	*C*(*C*^2^ − 2*S*^2^)	0	*C*(3*C*^2^ + 2*S*^2^)	0	*CS* ^2^	−*C*(*C*^2^ + 2*S*^2^)	0
*e*_f26_	0	−3*C*^2^*S*	0	−*C*^2^*S*	0	−*S*^3^	−*C*^2^*S*	0
*e*_f33_	*CS* ^3^	0	3*CS*^3^	0	*C* ^3^ *S*	0	0	*CS* ^3^
*e*_f34_	−*S*^2^(*S*^2^ − 3*C*^2^)	0	−3*S*^2^(*S*^2^ − 3*C*^2^)	0	−*C*^2^(3*S*^2^ − *C*^2^)	0	0	−*S*^2^(*S*^2^ − 3*C*^2^)
*e*_f35_	0	3*CS*^2^	0	*CS* ^2^	0	*C* ^3^	*CS* ^2^	0
*e*_f36_	0	−*S*(*S*^2^ − 2*C*^2^)	0	−*S*(3*S*^2^ + 2*C*^2^)	0	−*C*^2^*S*	*S*(*S*^2^ + 2*C*^2^)	0
*e*_f44_	2*CS*(*C*^2^ − *S*^2^)	0	6*CS*(*C*^2^ − *S*^2^)	0	−2*CS*(*C*^2^ − *S*^2^)	0	0	2*CS*(*C*^2^ − *S*^2^)
*e*_f45_	0	2*S*(2*C*^2^ − *S*^2^)	0	2*S*(2*C*^2^ + *S*^2^)	0	−2*C*^2^*S*	−2*S*^3^	0
*e*_f46_	0	−2*C*(2*S*^2^ − *C*^2^)	0	−2*C*(2*S*^2^ + *C*^2^)	0	2*CS*^2^	2*C*^3^	0
*e*_f55_	−2*CS*	0	2*CS*	0	0	0	0	2*CS*
*e*_f56_	−2(*C*^2^ − *S*^2^)	0	2(*C*^2^ − *S*^2^)	0	0	0	0	2(*C*^2^ − *S*^2^)
*e*_f66_	2*CS*	0	−2*CS*	0	0	0	0	−2*CS*

**Table 10:** Expansions of the matrices I to Q in terms of the unrotated *e*_fλμ_ Subscript f is the field direction. *C* and *S* stand for 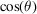 and 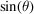, respectively, where θ is the facet angle.

	I	J	K	N	P	Q
*e*_f11_	3	1	0	0	0	1
*e*_f12_	2*C*^2^	6*C*^2^	*S* ^2^	0	0	−2*C*^2^
*e*_f13_	2*S*^2^	6*S*^2^	*C* ^2^	0	0	−2*S*^2^
*e*_f14_	4*CS*	12*CS*	−2*CS*	0	0	−4*CS*
*e*_f15_	0	0	0	0	0	0
*e*_f16_	0	0	0	0	0	0
*e*_f22_	3*C*^4^	*C* ^4^	*C* ^2^ *S* ^2^	*S* ^4^	*C* ^2^ *S* ^2^	*C* ^4^
*e*_f23_	6*C*^2^*S*^2^	2*C*^2^*S*^2^	*C*^4^ + *S*^4^	2*C*^2^*S*^2^	−2*C*^2^*S*^2^	2*C*^2^*S*^2^
*e*_f24_	12*C*^3^*S*	4*C*^3^*S*	−2*CS*(*C*^2^ − *S*^2^)	−4*CS*^3^	−2*CS*(*C*^2^ − *S*^2^)	4*C*^3^*S*
*e*_f25_	0	0	0	0	0	0
*e*_f26_	0	0	0	0	0	0
*e*_f33_	3*S*^4^	*S* ^4^	*C* ^2^ *S* ^2^	*C* ^4^	*C* ^2^ *S* ^2^	*S* ^4^
*e*_f34_	12*CS*^3^	4*CS*^3^	2*CS*(*C*^2^ − *S*^2^)	−4*C*^3^*S*	2*CS*(*C*^2^ − *S*^2^)	4*CS*^3^
*e*_f35_	0	0	0	0	0	0
*e*_f36_	0	0	0	0	0	0
*e*_f44_	12*C*^2^*S*^2^	4*C*^2^*S*^2^	−4*C*^2^*S*^2^	4*C*^2^*S*^2^	(*C*^2^ − *S*^2^)^2^	4*C*^2^*S*^2^
*e*_f45_	0	0	0	0	0	0
*e*_f46_	0	0	0	0	0	0
*e*_f55_	4*S*^2^	−4*S*^2^	0	0	*C* ^2^	4*S*^2^
*e*_f56_	8*CS*	−8*CS*	0	0	−2*CS*	8*CS*
*e*_f66_	4*C*^2^	−4*C*^2^	0	0	*S* ^2^	4*C*^2^

**Table:** This table is used in conjunction with Tables 12 ▸, 13 ▸ and 14 ▸; see text for details. For each of the nine 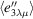, all *e*_2λμ_ components vanish for the *x*_2_ rotation. Apart from the multiplier, the *e*_1λμ_ and *e*_3λμ_ entries, for the *x*_2_ rotation, are identical for all 25 of the 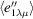, 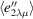 and 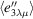.

*x*_2_ rotation	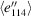	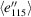	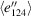	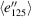	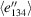	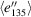	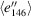	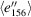
*e*_1λμ_ and *e*_3λμ_	d	c	b	a	f	e	h	g
*e*_2λμ_	a	b	c	d	e	f	g	h

**Table:** 

	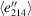	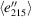	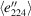	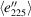	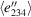	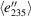	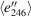	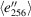
*e*_1λμ_ and *e*_3λμ_	a	b	c	d	e	f	g	h
e_2λμ_	d	c	b	a	f	e	h	g

**Table:** 

	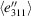	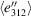	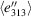	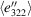	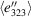	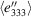	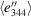	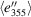	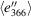
*e*_1λμ_ and e_3λμ_	i	j	k	i	k	n	p	p	q
*e*_2λμ_	Zero								

**Table:** See text for details. Subscript f is the field direction.

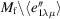	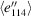	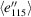	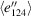	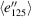	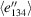	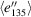	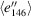	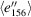
*M*_1_	*C*/8	*C*/8	*C*/8	*C*/8	*C*/2	*C*/2	−*C*/8	*C*/8
*M*_2_	−1/8	1/8	−1/8	1/8	−1/2	1/2	1/8	1/8
*M*_2_	−*S*/8	−*S*/8	−*S*/8	−*S*/8	−*S*/2	−*S*/2	*S*/8	−*S*/8

**Table:** 

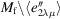	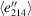	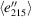	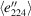	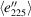	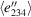	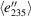	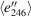	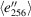
*M*_1_	*C*/8	−*C*/8	*C*/8	−*C*/8	*C*/2	−*C*/2	−*C*/8	−*C*/8
*M*_2_	1/8	1/8	1/8	1/8	1/2	1/2	−1/8	1/8
*M*_3_	−*S*/8	*S*/8	−*S*/8	*S*/8	−*S*/2	*S*/2	*S*/8	*S*/8

**Table:** 

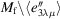	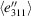	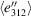	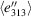	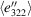	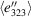	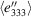	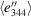	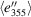	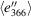
*M*_1_	*S*/8	*S*/8	*S*/2	*S*/8	*S*/2	*S*	*S*/2	*S*/2	*S*/8
*M*_2_	Zero								
*M*_3_	*C*/8	*C*/8	*C*/2	*C*/8	*C*/2	*C*	*C*/2	*C*/2	*C*/8

**Table 13:** Expansions of the matrices a to h in terms of the unrotated *e*_fλμ_ Subscript f is the field direction. *C* and *S* stand for 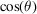 and 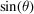, respectively, where θ is the facet angle.

	a	b	c	d	e	f	g	h
*e*_f11_	*C* ^3^ *S*	0	3*C*^3^*S*	0	*CS* ^3^	0	0	−*C*^3^*S*
*e*_f12_	3*CS*	0	*CS*	0	0	0	0	*CS*
*e*_f13_	−*CS*(*C*^2^ − *S*^2^)	0	−*CS*(*C*^2^ − *S*^2^)	0	*CS*(*C*^2^ − *S*^2^)	0	0	*CS*(*C*^2^ − *S*^2^)
*e*_f14_	0	*C*(*C*^2^ − 2*S*^2^)	0	*C*(3*C*^2^ + 2*S*^2^)	0	*CS* ^2^	−*C*(*C*^2^ + 2*S*^2^)	0
*e*_f15_	*C*^2^(*C*^2^ − 3*S*^2^)	0	3*C*^2^(*C*^2^ − 3*S*^2^)	0	−*S*^2^(*S*^2^ − 3*C*^2^)	0	0	−*C*^2^(*C*^2^ − 3*S*^2^)
*e*_f16_	0	3*C*^2^*S*	0	*C* ^2^ *S*	0	*S* ^3^	*C* ^2^ *S*	0
*e*_f22_	0	0	0	0	0	0	0	0
*e*_f23_	−3*CS*	0	−*CS*	0	0	0	0	−*CS*
*e*_f24_	0	3*C*	0	*C*	0	0	*C*	0
*e*_f25_	3(*C*^2^ − *S*^2^)	0	(*C*^2^ − *S*^2^)	0	0	0	0	(*C*^2^ − *S*^2^)
*e*_f26_	0	3*S*	0	*S*	0	0	*S*	0
*e*_f33_	−*CS*^3^	0	−3*CS*^3^	0	−*C*^3^*S*	0	0	*CS* ^3^
*e*_f34_	0	3*CS*^2^	0	*CS* ^2^	0	*C* ^3^	*CS* ^2^	0
*e*_f35_	−*S*^2^(*S*^2^ − 3*C*^2^)	0	−3*S*^2^(*S*^2^ − 3*C*^2^)	0	*C*^2^(*C*^2^ − 3*S*^2^)	0	0	*S*^2^(*S*^2^ − 3*C*^2^)
*e*_f36_	0	*S*(*S*^2^ − 2*C*^2^)	0	*S*(3*S*^2^ + 2*C*^2^)	0	*C* ^2^ *S*	−*S*(*S*^2^ + 2*C*^2^)	0
*e*_f44_	2*CS*	0	−2*CS*	0	0	0	0	2*CS*
*e*_f45_	0	2*S*(*S*^2^ − 2*C*^2^)	0	−2*S*(*S*^2^ + 2*C*^2^)	0	2*C*^2^*S*	2*S*^3^	0
*e*_f46_	−2(*C*^2^ − *S*^2^)	0	2(*C*^2^ − *S*^2^)	0	0	0	0	−2(*C*^2^ − *S*^2^)
*e*_f55_	−2*CS*(*C*^2^ − *S*^2^)	0	−6*CS*(*C*^2^ − *S*^2^)	0	2*CS*(*C*^2^ − *S*^2^)	0	0	2*CS*(*C*^2^ − *S*^2^)
*e*_f56_	0	2*C*(*C*^2^ − 2*S*^2^)	0	−2*C*(*C*^2^ + 2*S*^2^)	0	2*CS*^2^	2*C*^3^	0
*e*_f66_	−2*CS*	0	2*CS*	0	0	0	0	−2*CS*

**Table 14:** Expansions of the matrices i to q in terms of the unrotated *e*_fλμ_ Subscript f is the field direction. *C* and *S* stand for 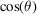 and 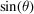, respectively, where θ is the facet angle.

	i	j	k	n	p	q
*e*_f11_	3*C*^4^	*C* ^4^	*C* ^2^ *S* ^2^	*S* ^4^	*C* ^2^ *S* ^2^	*C* ^4^
*e*_f12_	2*C*^2^	6*C*^2^	*S* ^2^	0	0	−2*C*^2^
*e*_f13_	6*C*^2^*S*^2^	2*C*^2^*S*^2^	(C^4^ + *S*^4^)	2*C*^2^*S*^2^	−2*C*^2^*S*^2^	2*C*^2^*S*^2^
*e*_f14_	0	0	0	0	0	0
*e*_f15_	−12*C*^3^*S*	−4*C*^3^*S*	2*CS*(*C*^2^ − *S*^2^)	4*CS*^3^	2*CS*(*C*^2^ − *S*^2^)	−4*C*^3^*S*
*e*_f16_	0	0	0	0	0	0
*e*_f22_	3	1	0	0	0	1
*e*_f23_	2*S*^2^	6*S*^2^	*C* ^2^	0	0	−2*S*^2^
*e*_f24_	0	0	0	0	0	0
*e*_f25_	−4*CS*	−12*CS*	2*CS*	0	0	4*CS*
*e*_f26_	0	0	0	0	0	0
*e*_f33_	3*S*^4^	*S* ^4^	*C* ^2^ *S* ^2^	*C* ^4^	*C* ^2^ *S* ^2^	*S* ^4^
*e*_f34_	0	0	0	0	0	0
*e*_f35_	−12*CS*^3^	−4*CS*^3^	−2*CS*(*C*^2^ − *S*^2^)	4*C*^3^*S*	−2*CS*(*C*^2^ −S^2^)	−4*CS*^3^
*e*_f36_	0	0	0	0	0	0
*e*_f44_	4*S*^2^	−4*S*^2^	0	0	*C* ^2^	4*S*^2^
*e*_f45_	0	0	0	0	0	0
*e*_f46_	−8*CS*	8*CS*	0	0	2*CS*	−8*CS*
*e*_f55_	12*C*^2^*S*^2^	4*C*^2^*S*^2^	−4*C*^2^*S*^2^	4*C*^2^*S*^2^	(*C*^2^ − *S*^2^)^2^	4*C*^2^*S*^2^
*e*_f56_	0	0	0	0	0	0
*e*_f66_	4*C*^2^	−4*C*^2^	0	0	*S* ^2^	4*C*^2^

**Table:** Results are identical for both *x*_1_ and *x*_2_ rotations. Table is in two parts, and is to be read as: 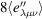 = 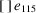 + 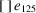 + 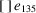
+ 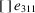 + 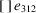 + 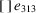 + 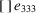 + 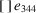.

	*e*_115_	*e*_125_	*e*_135_	*e*_311_
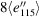	4*C*^3^(3*C*^2^ − 1)	0	12*C*^3^*S*^2^	−*CS*^2^(3*C*^2^ + 1)
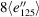	−4*C*^3^*S*^2^	8*C*^3^	4*C*^3^*S*^2^	4*CS*^4^
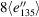	16*C*^3^*S*^2^	0	8*C*^3^(*C*^2^ − *S*^2^)	−4*CS*^4^
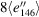	4*C*^5^	−4*C*^3^	4*C*^3^*S*^2^	−*CS*^2^(1 + *C*^2^)
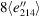	−4*C*^3^*S*^2^	8*C*^3^	4*C*^3^*S*^2^	*CS* ^4^
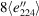	4*C*^3^(2*C*^2^ − *S*^2^)	0	12*C*^3^*S*^2^	−*C*(1 + 3*C*^2^)*S*^2^
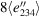	16*C*^3^*S*^2^	0	8*C*^3^(*C*^2^ − *S*^2^)	−4*CS*^4^
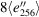	4*C*^5^	−4*C*^3^	4*C*^3^*S*^2^	*C*(*C*^4^ − 1)
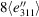	−4*CS*^2^(3*C*^2^ + 1)	0	−12*CS*^4^	*C*(3*C*^4^ + 2*C*^2^ + 3)
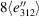	4*CS*^4^	−16*CS*^2^	−4*CS*^4^	*CS* ^4^
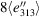	8*C*(*C*^2^ − *S*^2^)*S*^2^	8*CS*^2^	−8*C*(*C*^2^ − *S*^2^)*S*^2^	4*C*^3^*S*^2^
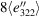	−4*C*(1 + 3*C*^2^)*S*^2^	0	−12*CS*^4^	*C*(3*C*^4^ + 2*C*^2^ + 3)
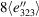	8*C*(*C*^2^ − *S*^2^)*S*^2^	8*CS*^2^	−8*C*(*C*^2^ − S^2^)*S*^2^	4*C*^3^*S*^2^
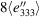	32*CS*^4^	0	32*C*^3^*S*^2^	8*CS*^4^
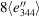	4*C*[1 + 2(*C*^2^ − *S*^2^)]*S*^2^	−4*CS*^2^	−8*C*(*C*^2^ − *S*^2^)*S*^2^	2*CS*^2^(2*C*^2^ + 1)
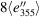	4*C*[1 + 2(*C*^2^ − *S*^2^)]*S*^2^	−4*CS*^2^	−8*C*(*C*^2^ − *S*^2^)*S*^2^	2*CS*^2^(2*C*^2^ + 1)
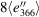	−4*C*(*C*^2^ + 1)*S*^2^	8*CS*^2^	−4*CS*^4^	*C*(*C*^2^ + 1)^2^

**Table:** 

	*e*_312_	*e*_313_	*e*_333_	*e*_344_
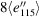	0	*CS*^2^[1 + 3(*C*^2^ − *S*^2^)]	3*CS*^4^	2*CS*^2^[1 + 3(*C*^2^ − *S*^2^)]
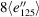	−4*CS*^2^	2*CS*^2^(*C*^2^ + 1)	*CS* ^4^	−4*CS*^4^
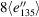	0	−4*CS*^2^(*C*^2^ − *S*^2^)	4*C*^3^*S*^2^	−8*CS*^2^(*C*^2^ − *S*^2^)
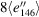	2*CS*^2^	−2*CS*^4^	*CS* ^4^	4*C*^3^*S*^2^
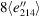	−4*CS*^2^	2*CS*^2^(*C*^2^ + 1)	*CS* ^4^	−4*CS*^4^
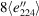	0	2*CS*^2^(3*C*^2^ − 1)	3*CS*^4^	4*CS*^2^(3*C*^2^ − 1)
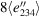	0	−4*CS*^2^(*C*^2^ − *S*^2^)	4*C*^3^*S*^2^	−8*CS*^2^(*C*^2^ − *S*^2^)
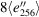	2*CS*^2^	−2*CS*^4^	*CS* ^4^	4*C*^3^*S*^2^
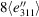	0	2*CS*^2^(3*C*^2^ + 1)	3*CS*^4^	4*CS*^2^(3*C*^2^ + 1)
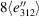	8*C*^3^	2*CS*^2^(*C*^2^ + 3)	*CS* ^4^	−4*CS*^4^
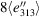	4*CS*^2^	4*C*(*C*^2^ + *C*^4^ + *S*^4^)	4*C*^3^*S*^2^	−16*C*^3^*S*^2^
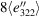	0	2*C*(3*C*^2^ + 1)*S*^2^	3*CS*^4^	4*C*(1 + 3*C*^2^)*S*^2^
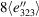	4*CS*^2^	4*C*(*C*^2^ + *C*^4^ + *S*^4^)	4*C*^3^*S*^2^	−16*C*^3^*S*^2^
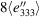	0	16*C*^3^*S*^2^	8*C*^5^	32*C*^3^*S*^2^
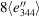	−2*CS*^2^	−8*C*^3^*S*^2^	4*C*^3^*S*^2^	4*C*[*C*^2^ + (*C*^2^ − *S*^2^)^2^]
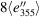	−2*CS*^2^	−8*C*^3^*S*^2^	4*C*^3^*S*^2^	4*C*[*C*^2^ + (*C*^2^ − *S*^2^)^2^]
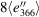	−4*C*^3^	−2*CS*^4^	*CS* ^4^	4*C*(*C*^2^ + 1)*S*^2^

**Table 16:** Relations among the rank 6 elastic coefficients for Laue groups Va, Vb, VIa and VIb and for isotropy Lowercase letters in parentheses correspond to those in Table 3 of Brugger (1965 ▸).

Laue Va, Vb, VIa and VIb		
(a): *c*_122_ = (*c*_111_ + *c*_112_ − *c*_222_)	(b): 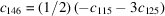	(c): 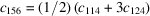
(d): 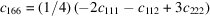	(e): 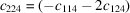	(f): 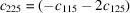
(g): 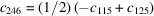	(h): 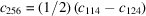	(i): 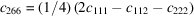
(j): 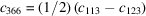	(k): 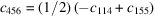	

Isotropy		
(l): 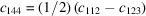	(m): 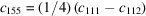	(n): 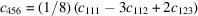

**Table 17:** Number of independent second-, third-, fourth- and fifth-order elastic coefficients for all Laue classes The second-order results are given, *e.g.* in IEEE Standard on Piezoelectricity (1987 ▸), Cady (1946 ▸), Nye (1985 ▸). Those of the third order are given by Fumi (1951 ▸), Brugger (1965 ▸). Krishnamurty (1963 ▸) provides the fourth order, and those of the fifth order are found in Krishnamurty & Gopalakrishnamurty (1968 ▸). For isotropy, the number of independent coefficients is equal to one-half the tensor rank.

Laue →	I	II	III	IVa	IVb	Va	Vb	VIa	VIb	VIIa	VIIb	Isotropy
Rank ↓	Number of independent coefficients											
4	21	13	9	7	6	7	6	5	5	3	3	2
6	56	32	20	16	12	20	14	12	10	8	6	3
8	126	70	42	36	25	42	28	24	19	14	11	4
10	252	136	78	68	44	84	52	46	33	26	18	5

**Table 18:** Indices of the 24 finite, averaged, elastic stiffnesses 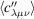 for triclinic Laue group I

111	112	113	122	123	133	144	145
155	166	222	223	233	244	245	255
266	333	344	355	366	446	456	556

**Table 19:** Number of rank 6 elastic coefficients for all Laue groups The row labeled ‘total’ lists the number of 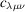 entries; the row below it lists the number of these that are independent. The last row gives the number of finite 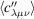 entries. In Laue groups I, II, IVa, Va and VIa all of the 24 entries enumerated in Table 18 ▸ exist. In Laue groups III, IVb, Vb, VIb, VIIa and VIIb the relations 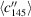 = 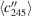 = 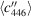 = 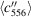

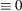
 reduce the number of finite entries to 20. In Laue groups VIb and VIIb the following equalities occur: 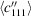 = 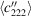, 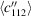 = 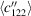, 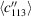 = 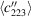, 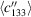 = 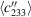, 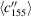 = 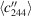, 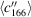 = 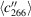, and 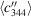 = 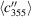, further reducing the number of relations to 13; see Tables 44 ▸ and 45 ▸. For isotropic substances, all 20 finite 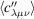 are obviously equal to the corresponding unrotated 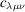 values.

System →	Triclinic	Monoclinic	Rhombic	Tetragonal		Trigonal		Hexagonal		Cubic		Isotropic
Laue →	I	II	III	IVa	IVb	Va	Vb	VIa	VIb	VIIa	VIIb	(VIII)
Total	56	32	20	28	20	50	31	28	20	20	20	20
Independent	56	32	20	16	12	20	14	12	10	8	6	3
Finite 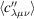	24	24	20	24	20	24	20	24	20	20	20	20

**Table 20:** Expansion of rank 6, *x*_1_ rotation, Laue group I (triclinic) averaged elastic stiffness 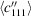 The expansions in Tables 20 ▸ to 43 ▸ are to be read as: 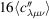 = 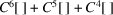 + 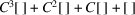. *C* and *S* are cos(θ) and sin(θ), respectively, where θ is the facet angle.

*C* ^6^	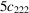
*C* ^5^	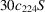
*C* ^4^	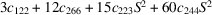
*C* ^3^	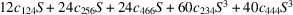
*C* ^2^	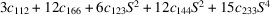 + 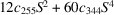 + 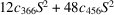
*C* ^1^	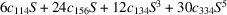 + 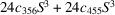
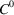	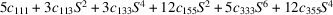

**Table 21:** Expansion of rank 6, *x*_1_ rotation, Laue group I (triclinic) averaged elastic stiffness 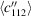 See expansion rule in caption to Table 20 ▸.

*C* ^6^	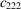
*C* ^5^	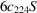
*C* ^4^	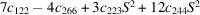
*C* ^3^	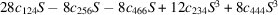
*C* ^2^	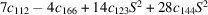 + 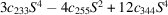 − 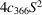 − 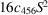
*C* ^1^	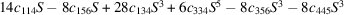
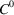	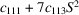 + 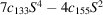 + 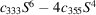

**Table 22:** Expansion of rank 6, *x*_1_ rotation, Laue group I (triclinic) averaged elastic stiffness 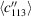 See the expansion rule in caption to Table 20 ▸.

*C* ^6^	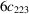
*C* ^5^	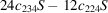
*C* ^4^	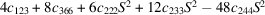 + 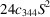
*C* ^3^	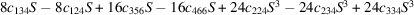 − 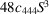
*C* ^2^	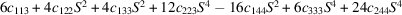 − 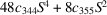 + 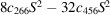
*C* ^1^	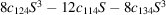 + 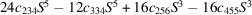
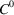	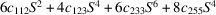

**Table 23:** Expansion of rank 6, *x*_1_ rotation, Laue group I (triclinic) averaged elastic stiffness 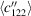 See the expansion rule in caption to Table 20 ▸.

*C* ^6^	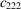
*C* ^5^	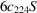
*C* ^4^	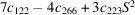 + 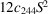
*C* ^3^	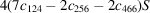 + 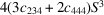
*C* ^2^	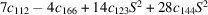 + 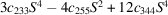 − 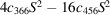
*C* ^1^	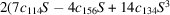 + 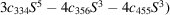
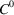	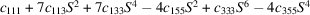

**Table 24:** Expansion of rank 6, *x*_1_ rotation, Laue group I (triclinic) averaged elastic stiffness 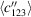 See the expansion rule in caption to Table 20 ▸.

*C* ^6^	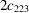
*C* ^5^	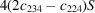
*C* ^4^	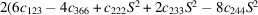 + 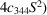
*C* ^3^	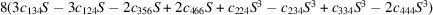
*C* ^2^	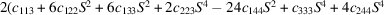 − 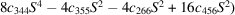
*C* ^1^	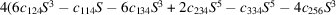 + 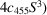
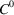	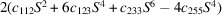

**Table 25:** Expansion of rank 6, *x*_1_ rotation, Laue group I (triclinic) averaged elastic stiffness 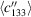 See the expansion rule in caption to Table 20 ▸.

*C* ^6^	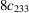
*C* ^5^	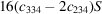
*C* ^4^	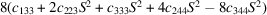
*C* ^3^	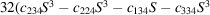 + 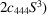
*C* ^2^	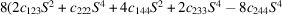 + 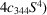
*C* ^1^	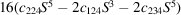
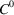	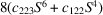

**Table 26:** Expansion of rank 6, *x*_1_ rotation, Laue group I (triclinic) averaged elastic stiffness 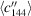 See the expansion rule in caption to Table 20 ▸.

*C* ^6^	2*c*_244_
*C* ^5^	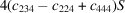
*C* ^4^	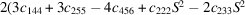 + 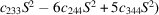
*C* ^3^	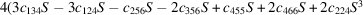 − 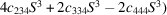
*C* ^2^	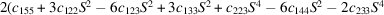 + 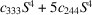 + 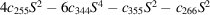 + 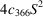 − 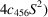
*C* ^1^	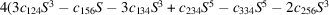 + 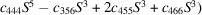
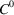	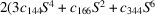 + 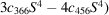

**Table 27:** Expansion of rank 6, *x*_1_ rotation, Laue group I (triclinic) averaged elastic stiffness 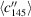 See the expansion rule in caption to Table 20 ▸.

*C* ^6^	0
*C* ^5^	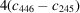
*C* ^4^	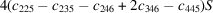
*C* ^3^	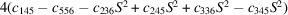
*C* ^2^	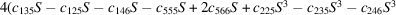 + 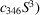
*C* ^1^	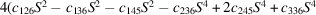 − 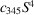 − 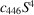 + 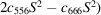
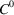	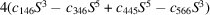

**Table 28:** Expansion of rank 6, *x*_1_ rotation, Laue group I (triclinic) averaged elastic stiffness 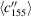 See the expansion rule in caption to Table 20 ▸.

*C* ^6^	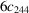
*C* ^5^	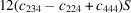
*C* ^4^	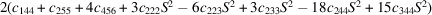
*C* ^3^	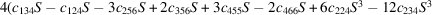 + 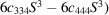
*C* ^2^	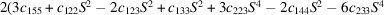 + 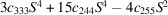 − 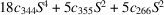 − 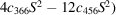
*C* ^1^	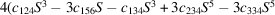 + 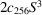 + 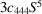 − 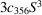 − 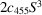 + 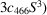
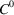	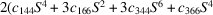 + 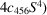

**Table 29:** Expansion of rank 6, *x*_1_ rotation, Laue group I (triclinic) averaged elastic stiffness 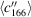 See the expansion rule in caption to Table 20 ▸.

*C* ^6^	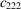
*C* ^5^	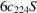
*C* ^4^	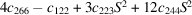
*C* ^3^	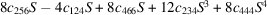
*C* ^2^	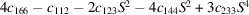 + 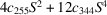 + 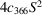 + 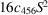
*C* ^1^	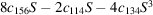 + 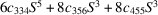
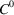	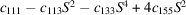 + 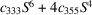

**Table 30:** Expansion of rank 6, *x*_1_ rotation, Laue group I (triclinic) averaged elastic stiffness 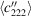 See the expansion rule in caption to Table 20 ▸.

*C* ^6^	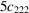
*C* ^5^	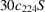
*C* ^4^	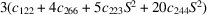
*C* ^3^	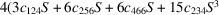 + 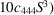
*C* ^2^	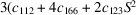 + 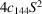 + 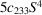 + 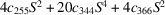 + 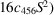
*C* ^1^	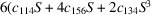 + 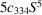 + 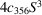 + 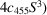
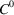	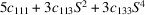 + 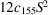 + 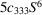 + 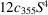

**Table 31:** Expansion of rank 6, *x*_1_ rotation, Laue group I (triclinic) averaged elastic stiffness 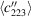 See the expansion rule in caption to Table 20 ▸.

*C* ^6^	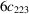
*C* ^5^	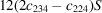
*C* ^4^	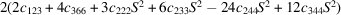
*C* ^3^	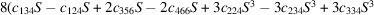 − 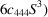
*C* ^2^	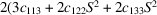 + 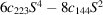 + 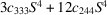 − 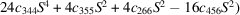
*C* ^1^	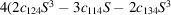 + 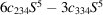 + 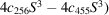
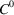	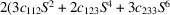 + 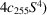

**Table 32:** Expansion of rank 6, *x*_1_ rotation, Laue group I (triclinic) averaged elastic stiffness 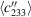 See the expansion rule in caption to Table 20 ▸.

*C* ^6^	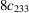
*C* ^5^	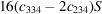
*C* ^4^	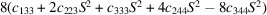
*C* ^3^	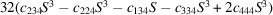
*C* ^2^	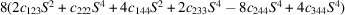
*C* ^1^	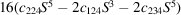
*C* ^0^	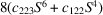

**Table 33:** Expansion of rank 6, *x*_1_ rotation, Laue group I (triclinic) averaged elastic stiffness 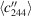 See the expansion rule in caption to Table 20 ▸.

*C* ^6^	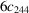
*C* ^5^	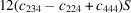
*C* ^4^	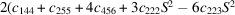 + 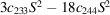 + 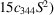
*C* ^3^	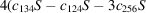 + 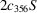 + 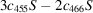 + 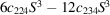 + 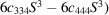
*C* ^2^	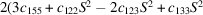 + 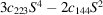 − 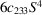 + 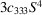 + 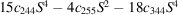 + 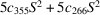 − 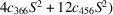
*C* ^1^	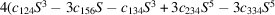 + 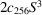 + 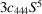 − 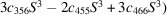
*C* ^0^	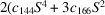 + 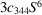 + 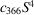 + 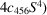

**Table 34:** Expansion of rank 6, *x*_1_ rotation, Laue group I (triclinic) averaged elastic stiffness 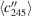 See the expansion rule in caption to Table 20 ▸.

*C* ^6^	0
*C* ^5^	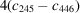
*C* ^4^	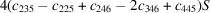
*C* ^3^	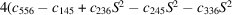 + 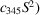
*C* ^2^	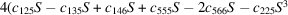 + 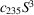 + 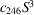 − 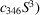
*C* ^1^	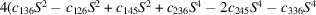 + 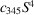 + 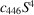 − 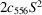 + 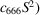
*C* ^0^	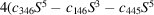 + 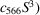

**Table 35:** Expansion of rank 6, *x*_1_ rotation, Laue group I (triclinic) averaged elastic stiffness 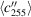 See the expansion rule in caption to Table 20 ▸.

*C* ^6^	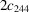
*C* ^5^	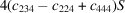
*C* ^4^	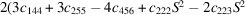 + 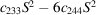 + 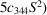
*C* ^3^	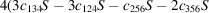 + 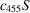 + 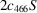 + 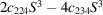 + 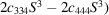
*C* ^2^	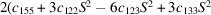 + 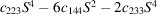 + 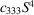 + 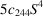 + 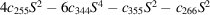 + 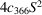 − 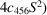
*C* ^1^	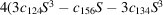 + 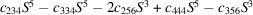 + 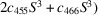
*C* ^0^	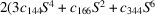 + 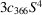 + 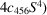

**Table 36:** Expansion of rank 6, *x*_1_ rotation, Laue group I (triclinic) averaged elastic stiffness 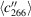 See the expansion rule in caption to Table 20 ▸.

*C* ^6^	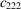
*C* ^5^	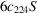
*C* ^4^	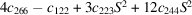
*C* ^3^	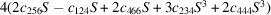
*C* ^2^	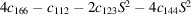 + 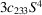 + 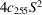 + 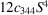 + 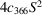 + 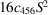
*C* ^1^	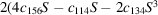 + 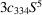 + 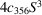 + 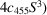
*C* ^0^	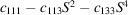 + 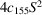 + 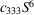 + 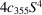

**Table 37:** Expansion of rank 6, *x*_1_ rotation, Laue group I (triclinic) averaged elastic stiffness 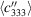 See the expansion rule in caption to Table 20 ▸.

*C* ^6^	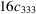
*C* ^5^	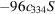
*C* ^4^	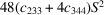
*C* ^3^	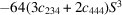
*C* ^2^	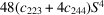
*C* ^1^	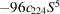
*C* ^0^	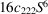

**Table 38:** Expansion of rank 6, *x*_1_ rotation, Laue group I (triclinic) averaged elastic stiffness 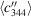 See the expansion rule in caption to Table 20 ▸.

*C* ^6^	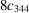
*C* ^5^	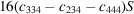
*C* ^4^	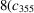 + 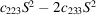 + 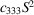 + 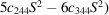
*C* ^3^	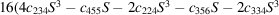 + 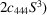
*C* ^2^	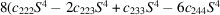 + 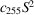 + 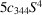 + 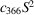 + 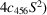
*C* ^1^	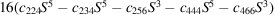
*C* ^0^	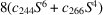

**Table 39:** Expansion of rank 6, *x*_1_ rotation, Laue group I (triclinic) averaged elastic stiffness 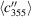 See the expansion rule in caption to Table 20 ▸.

*C* ^6^	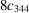
*C* ^5^	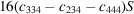
*C* ^4^	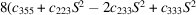 + 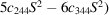
*C* ^3^	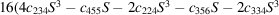 + 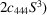
*C* ^2^	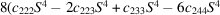 + 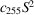 + 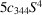 + 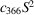 + 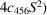
*C* ^1^	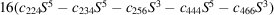
*C* ^0^	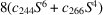

**Table 40:** Expansion of rank 6, *x*_1_ rotation, Laue group I (triclinic) averaged elastic stiffness 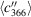 See the expansion rule in caption to Table 20 ▸.

*C* ^6^	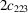
*C* ^5^	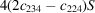
*C* ^4^	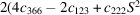 + 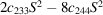 + 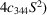
*C* ^3^	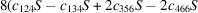 + 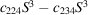 + 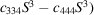
*C* ^2^	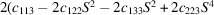 + 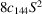 + 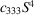 + 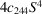 − 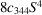 + 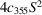 + 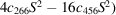
*C* ^1^	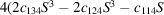 + 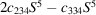 + 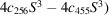
*C* ^0^	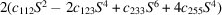

**Table 41:** Expansion of rank 6, *x*_1_ rotation, Laue group I (triclinic) averaged elastic stiffness 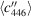 See the expansion rule in caption to Table 20 ▸.

*C* ^6^	0
*C* ^5^	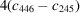
*C* ^4^	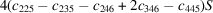
*C* ^3^	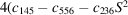 + 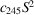 + 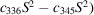
*C* ^2^	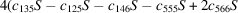 + 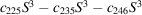 + 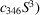
*C* ^1^	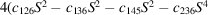 + 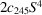 + 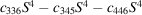 + 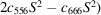
*C* ^0^	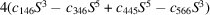

**Table 42:** Expansion of rank 6, *x*_1_ rotation, Laue group I (triclinic) averaged elastic stiffness 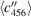 See the expansion rule in caption to Table 20 ▸.

*C* ^6^	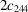
*C* ^5^	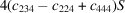
*C* ^4^	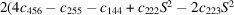 + 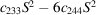 + 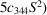
*C* ^3^	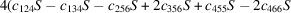 + 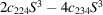 + 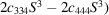
*C* ^2^	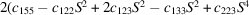 + 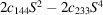 + 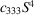 + 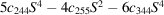 + 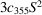 + 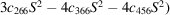
*C* ^1^	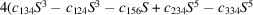 + 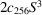 + 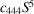 − 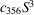 − 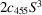 + 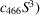
*C* ^0^	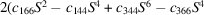 + 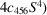

**Table 43:** Expansion of rank 6, *x*_1_ rotation, Laue group I (triclinic) averaged elastic stiffness 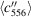 See the expansion rule in caption to Table 20 ▸.

*C* ^6^	0
*C* ^5^	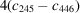
*C* ^4^	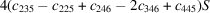
*C* ^3^	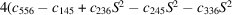 + 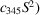
*C* ^2^	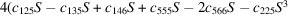 + 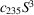 + 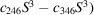
*C* ^1^	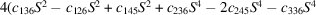 + 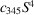 + 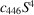 − 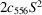 + 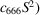
*C* ^0^	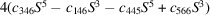

**Table:** Entries are to be divided by 16, except 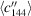, 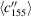 = 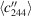, 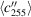 and 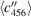; these are to be divided by 32. For typographical clarity, the table is divided into two parts; see text for details.

	*c*_111_	*c*_112_	*c*_113_	*c*_123_	*c*_133_
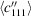 = 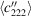	0 / 9 / −6 / 5	0	−15 / 9 / 3 / 3	0	15 / −27 / 9 / 3
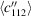 = 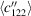	0 / 5 / 2 / 1	0 / 8 / 8 / 0	−3 / 5 / −9 / 7	0 /−16 / 16 / 0	3 / 1 / −11 / 7
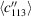 = 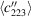	0 / −8 / 8 / 0	0 /−2 / −4 / 6	18 / −20 / 18 / 0	0 / 4 / −8 / 4	−18 / 26 / −14 / 6
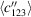	0 / −8 / 8 / 0	0 / −14 / 12 / 2	6 / −12 / 6 / 0	0 / 28 / −24 / 12	−6 / −2 / 6 / 2
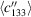 = 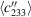	0 / 8 / −16 / 8	0 / 8 / −16 / 8	−24 / 40 / −24 / 8	0 / −16 / 16 / 0	24 / −24 / 16 / 0
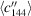	0 / −10 / 12 / −2	0 / −13 / 14 / −1	12 / −18 / 0 / 6	0 / 26 / −20 / −6	−12 / −8 / 4 / 0
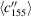 = 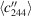	0 / −14 / 20 / −6	0 / 1 / 2 / −3	36 / −38 / 0 / 2	0 / −2 / 4 / −2	−36 / 56 / −20 / 0
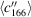 = 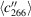	0 / 1 / −2 / 1	0 / −2 / −2 / 0	−3 / 1 / 3 / −1	0 / 4 / −4 / 0	3 / −7 / 5 / −1
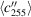	0 / −10 / 12 / −2	0 / −13 / 14 / −1	12 / −18 / 0 / 6	0 / 26 / −20 / −6	−12 / 8 / 4 / 0
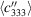	0	0	48 / −96 / 48 / 0	0	−48 / 48 / 0 / 0
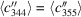	0 / 4 / −8 / 4	0 / −2 / 4 / −2	−24 / 36 / −12 / 0	0 / 4 / −4 / 0	24 / −32 / 8 / 0
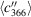	0	0 / 6 / −8 / 2	6 / −4 / 6 / 0	0 / −12 / 8 / −4	−6 / 14 / −10 / 2
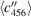	0 / −2 / 4 / −2	0 / 7 / −6 / −1	12 / −10 / 0 / −2	0 / −14 / 12 / 2	−12 / 24 / −12 / 0

**Table:** 

	*c*_144_	*c*_155_	*c*_222_	*c*_333_	*c*_344_
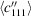 = 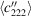	0	−60 / 36 / 12 / 12	5 / −6 / 9 / 0	−5 / 15 / −15 / 5	60 / −108 / 36 / 12
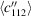 = 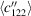	0 / −32 / 32 / 0	−12 / 20 / −4 / −4	1 / −6 / −3 / 0	−1 / 3 / −3 / 1	12 / −28 / 20 / −4
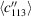 = 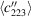	0 / 8 / −16 / 8	72 / −80 / 8 / 0	−6 / 12 / −6 / 0	6 / −12 / 6 / 0	−72 / 112 / −40 / 0
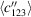	0 / 56 / −48 / −8	24 / −48 / 24 / 0	−2 / 12 / −10 / 0	2 / −4 / 2 / 0	−24 / 48 / −24 / 0
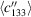 = 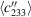	0 / −32 / 32 / 0	−96 / 160 / −64 / 0	8 / −24 / 24 / −8	−8 / 8 / 0 / 0	96 / −128 / 32 / 0
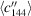	0 / 52 / −40 / 20	48 / −72 / 32 / −8	−4 / 15 / −14 / 3	4 / −8 / 4 / 0	−48 / 84 / −40 / 4
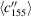 = 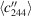	0 / −4 / 8 / −4	144 / −152 / 32 / 8	−12 / 21 / −18 / 9	12 / −24 / 12 / 0	−144 / 220 / −88 / 12
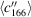 = 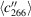	0 / 8 / −8 / 0	−12 / 4 / 4 / 4	1 / 0 / 3 / 0	−1 / 3 / −3 / 1	12 / −20 / 4 / 4
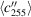	0 / 52 / −40 / 20	48 / −72 / 32 / −8	−4 / 15 / −14 / 3	4 / −8 / 4 / 0	−48 / 84 / −40 / 4
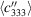	0	192 / −384 / 192 / 0	−16 / 48 / −48 / 16	16 / 0 / 0 / 0	−192 / 192 / 0 / 0
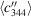 = 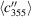	0 / 8 / −8 / 0	−96 / 144 / −56 / 8	8 / −18 / 12 / −2	−8 / 8 / 0 / 0	96 / −120 / 40 / 0
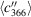	0 / −24 / 16 / 8	24 / −16 / −8 / 0	−2 / 0 / 2 / 0	2 / −4 / 2 / 0	−24 / 32 / −8 / 0
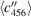	0 / −28 / 24 / −12	48 / −40 / 0 / 8	−4 / 3 / −2 / 3	4 / −8 / 4 / 0	−48 / 68 / −24 / 4

**Table 45:** Cubic, VIIb, class 43*m* 〈*c*′′〉 coefficients, *x*_1_ rotation All 〈*c*′′〉 entries are to be divided by 16. The entries are to be read as, *e.g.*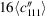 = 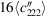 = (10 − 15*C*^2^*S*^2^)*c*_111_ + (6 + 9*C*^2^*S*^2^)*c*_112_ + … + (24 + 36*C*^2^*S*^2^)*c*_155_ + (48*C*^2^*S*^2^)*c*_456_, where *C* is cos(θ), and *S* is sin(θ), and θ is the facet angle.

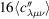	*c*_111_	*c*_112_	*c*_123_	*c*_144_	*c*_155_	*c*_456_
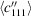 = 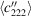	10 / −15	6 / 9	0 / 6	0 / 36	24 / 36	0 / 48
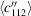 = 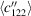	2 / −3	14 / −11	0 / 14	0 / 20	−8 / 20	0 / −16
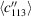 = 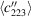	0 / 6	12 / 2	4 / −8	8 / −32	0 / −8	0 / −32
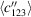	0 / 2	4 / 22	12 / −24	−8 / −32	0 / −24	0 / 32
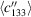 = 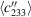	0 / 8	16 / −24	0 / 16	0 / 32	0 / −32	0 / 0
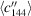	0 / 2	0 / 10	0 / −12	12 / −20	4 / −12	−8 / 8
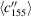 = 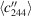	0 / 6	0 / −2	0 / −4	4 / −28	12 / −4	8 / −40
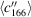 = 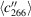	2 / −3	−2 / 5	0 / −2	0 / 4	8 / 4	0 / 16
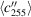	0 / 2	0 / 10	0 / −12	12 / −20	4 / −12	−8 / 8
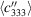	16 / −48	0 / 48	0 / 0	0 / 0	0 / 192	0 / 0
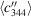 = 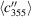	0 / 8	0 / −8	0 / 0	0 / 16	16 / −48	0 / 32
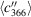	0 / 2	4 / −10	−4 / 8	8 / 0	0 / 8	0 / −32
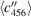	0 / 2	0 / −6	0 / 4	−4 / −4	4 / 4	8 / −24

**Table 46:** Linear pyroelectric coefficients (*p*_*j*_) referred to the unrotated crystal axes

System →	Triclinic	Monoclinic		Rhombic	Tetragonal		Trigonal		Hexagonal	
Laue →	I	II		III	IVa	IVb	Va	Vb	VIa	VIb
H-M →	1	2	*m*	*mm*2	4	4*mm*	3	3*m*	6	6*mm*
	Pyroelectric matrix									
	*p*_1_	0	*p*_1_	0						
	*p*_2_	*p*_2_	0	0						
	*p*_3_	0	*p*_3_	*p*_3_						

**Table 47:** Averaged linear pyroelectric coefficients for both *x*_1_ and *x*_2_ rotations The averaged 〈*p*_1_〉 and 〈*p*_2_〉 coefficients vanish identically: 〈*p*_1_〉 = 〈*p*_2_〉 ≡ 0.

System →	Triclinic	Monoclinic		Rhombic	Tetragonal		Trigonal		Hexagonal	
Laue →	I	II		III	IVa	IVb	Va	Vb	VIa	VIb
H-M →	1	2	*m*	*mm*2	4	4*mm*	3	3*m*	6	6*mm*
Rotation	〈*p*_3_〉									
*x*_1_	−*p*_2_*S*	−*p*_2_*S*	0	+*p*_3_*C*						
	+*p*_3_*C*									
*x*_2_	+*p*_1_*S*	0	+*p*_1_*S*	+*p*_3_*C*						
	+*p*_3_*C*		+*p*_3_*C*							
